# The Green Tea Catechin Epigallocatechin Gallate (EGCG) Blocks Cell Motility, Chemotaxis and Development in *Dictyostelium discoideum*

**DOI:** 10.1371/journal.pone.0059275

**Published:** 2013-03-14

**Authors:** Kyle J. McQuade, Akihiko Nakajima, April N. Ilacqua, Nao Shimada, Satoshi Sawai

**Affiliations:** 1 Department of Biological Sciences, Colorado Mesa University, Grand Junction, Colorado, United States of America; 2 Graduate School of Arts and Sciences, The University of Tokyo, Tokyo, Japan; 3 Research Center for Complex Systems Biology, The University of Tokyo, Tokyo, Japan; 4 PRESTO, Japan Science and Technology Agency, Tokyo, Japan; Cardiff University, United Kingdom

## Abstract

Catechins, flavanols found at high levels in green tea, have received significant attention due to their potential health benefits related to cancer, autoimmunity and metabolic disease, but little is known about the mechanisms by which these compounds affect cellular behavior. Here, we assess whether the model organism *Dictyostelium discoideum* is a useful tool with which to characterize the effects of catechins. Epigallocatechin gallate (EGCG), the most abundant and potent catechin in green tea, has significant effects on the *Dictyostelium* life cycle. In the presence of EGCG aggregation is delayed, cells do not stream and development is typically stalled at the loose aggregate stage. The developmental effects very likely result from defects in motility, as EGCG reduces both random movement and chemotaxis of *Dictyostelium* amoebae. These results suggest that catechins and their derivatives may be useful tools with which to better understand cell motility and development in *Dictyostelium* and that this organism is a useful model to further characterize the activities of catechins.

## Introduction

Catechins, plant secondary metabolites that are found at high levels in green and black teas and dark chocolate, have attracted much attention due to their health-promoting characteristics. Accumulating evidence suggests that these compounds modulate the immune system [1], increase metabolic rate [2], reduce atherosclerotic lesions [3] and protect against cognitive impairment [4]. Additional research has been aimed at understanding the effects of catechins on tumor growth with recent observational studies suggesting that consumption of catechin-rich green tea may help prevent breast, prostate and other cancers [5]–[6]. Individual catechins or polyphenon E, a pharmaceutical-grade mixture containing several catechins, have also been tested in numerous preclinical and clinical trials, where they have been shown to have chemopreventive and chemotherapeutic activities [7]–[9]. Many recent studies suggest that catechins exert their effects via a variety of mechanisms including but not limited to acting as antioxidants, modulators of cellular signaling pathways and inhibitors of chromatin remodeling enzymes, but the precise mechanisms by which these compounds might provide health benefits are not yet clear [10].

The social amoeba *Dictyostelium discoideum* has been used to model a number of human diseases including neurological disorders [11], bacterial infection [12] and mitochondrial disease [13], and social amoebae are being used increasingly to characterize the activities of small molecules [14]. The organism exhibits a remarkable life cycle in which nutrient-deprived amoebae migrate together to form multicellular aggregates, differentiate into specialized cell types and form fruiting bodies in which groups of encapsulated spores are suspended atop cellulosic stalks. Because the signaling mechanisms amoebae use to communicate, aggregate and differentiate parallel those that commonly malfunction in human diseases, *Dictyostelium* is a useful surrogate with which to analyze effectiveness of potential pharmaceuticals. Unlike traditional screening methods that require assessment of specific, individual outcomes, multiple outcomes can be assessed simultaneously by monitoring the *Dictyostelium* life cycle [15]. Furthermore, because *Dictyostelium* has been the focus of extensive research, several robust methods are available to characterize and quantify aggregation, differentiation and morphogenesis that make up the *Dictyostelium* life cycle. Finally, because many of the steps in the life cycle have been characterized in significant detail, the particular stage that is affected by a test compound may provide clues about molecular targets.

Although *Dictyostelium* has been used to characterize the activities of bisphosphonates [16], lithium drugs [17], chemotherapies [18] and teratogens [19]–[20], this organism has not been routinely used to characterize the effects of catechins or other natural products. In this study, we investigate the effects of catechins, and especially epigallocatechin gallate (EGCG), on the *Dictyostelium* life cycle. EGCG alters aggregation of starving cells, and the aggregates that form fail to form slugs. These effects are accompanied by delayed expression of developmentally-regulated genes and defects in chemotaxis. These results confirm that *Dictyostelium* is a useful system in which to characterize the biological activities of catechins and suggest that catechins may be useful tools to characterize cell motility and development in this organism.

## Methods

### Cell Growth and Development

The *Dictyostelium* strain A×4 was cultured on lawns of *Klebsiella aerogenes* or grown in shaking axenic culture in HL-5 medium following standard procedures. For developmental experiments, thin layers of agar were prepared by pouring 0.6 mL of 1% agar in KK2 buffer in 3.8 cm^2^ wells. Test compounds were added by overlaying solidified wells with 0.6 mL KK2 containing twice the desired concentration and incubated at room temperature for 18–36 h. Compounds were obtained from Sigma (catechins, gallic acid, methyl 3,4,5-trihydroxybenzoic acid and cAMP). The overlay solution was decanted and the surface allowed to dry before adding 7×10^6^ cells per well. In some experiments, amoebae were collected by scraping the feeding front of plaques and placed directly on agar. For cAMP pulsing experiments, cells that were grown in shaking suspension were rinsed and resuspended at 5×10^6^ cells/mL in KK2 and starved with shaking at 22°C for 1 h before being pulsed with 50 nM cAMP every 6 minutes for 5 h. Aggregation, development and cell viability were documented using a Leica DMB6000 inverted microscope equipped with phase contrast and fluorescence optics and a Hamamatsu ORCA-03G CCD. Some images were acquired with a Leica M165C stereomicroscope equipped with a Leica DFC295 digital camera and Leica Montage for extended focusing. Digital images were processed in Adobe Photoshop to enhance levels and contrast.

For wave observations, A×4 cells expressing the cAMP FRET sensor epac1-camps were axenically grown in a modified HL-5 medium supplemented with 10 μg/ml G418. Agar plates were prepared by pouring 1.4 mL of agar (1% Bacto agar in KK2) in 9.5 cm^2^ wells (6 well cell culture plate (3506), Corning) for dark-field imaging. For FRET experiments, 2.8 mL of the agar solution was poured in a 60 mm dish (60 mm/non-treated dish (1010-060), IWAKI). EGCG was added on solidified agar wells by overlaying 1.4 mL (for dark-field imaging) or 2.8 mL (for FRET imaging) KK2 containing twice the desired concentration and allowed to equilibrate at room temperature overnight (14–20 h). The overlay solution was decanted and the surface was allowed to dry before plating the cells. Growing cells were washed twice and resuspended in KK2 at 1×10^7^ cells/mL. One milliliter of the cell suspension was deposited on the 1% agar plates with or without 3 mM EGCG. The supernatants were removed after 15 minutes, and the plates were dried for additional 15 minutes in a sterile hood. For FRET measurements, the plates were incubated at 22°C for 3–4 h prior to timelapse imaging.

### Imaging and Analysis of cAMP Signaling

Cells washed free of nutrients were plated at a monolayer cell density on 1% agar with or without EGCG. Acquisition of optical density waves was performed using custom-built darkfield optics as described earlier [21]–[22]. In this study, a digital CMOS camera (IEEE1394, A741; PixeLINK) equipped with a macro lens (25 mm f1.4; Computar) was used to acquire 480×480 pixel 8-bit grey-scale images every 30 seconds for 15 hours. For FRET-based cAMP measurements, fluorescent intensity of CFP and YFP from A×4 cells expressing the cAMP sensor epac1-camps [23] was acquired using a motorized inverted microscope (IX81; Olympus) equipped with an EM-CCD camera (QuantEM 512SC; Photometrics). A 10x air objective lens (0.4 NA, UplanSApo; Olympus), 435 nm excitation filter (BP425–445HQ; Olympus) and 460–510 nm (BA460–510HQ; Olympus) and 515–560 nm (BA515–560HQ; Olympus) bandpass filters were used to detect fluorescence from CFP and YFP respectively. A dichroic mirror (DM450; Olympus) was used to separate emission from excitation. 16-bit images were acquired from 6.7 h (400 min) after plating at 30 sec intervals for 10 h. Images were stored as TIFF files and later analyzed using ImageJ. The fluorescence ratio of CFP/YFP was calculated as an indicator for the level of cytosolic cAMP.

### Chemotaxis Assays

For chemotaxis experiments, washed cells were suspended at 5×10^6^ cells/ml in DB, starved for one hour by shaking at 155 rpm and subsequently pulsed with 50 nM cAMP for another 3 or 6 h. Chemotaxis was observed in spatial gradients of cAMP formed in a Dunn chamber (DCC100, Hawksley). For control experiments, the inner well of the chamber was filled with DB containing 2 mM caffeine, and the outer well was filled with DB containing 2 mM caffeine and 1 μM cAMP. For catechin treatment, the inner well was filled with 3 mM EGCG and 2 mM caffeine solution and the outer well was filled with solution containing 3 mM EGCG, 2 mM caffeine and 1 μM cAMP. For the analysis of basic motility without cAMP, starved cells suspended in 2 mM caffeine solution were plated onto a glass bottom dish (MatTek, Ashland, MA) and let stand for 30 min before adding caffeine and EGCG to the final concentrations used in Dunn chamber experiments. Experiments were repeated three times.

Images were obtained with a 20x objective and recorded every 6 seconds for 15 minutes. Cells were tracked for 5 to 15 minutes. Cell tracking and calculations of chemotaxis properties (speed, directionality, upward directionality, directional change and roundness) [24]–[26] were done with DYNAMIK software [27] and custom programs written in MATLAB (The MathWorks, Natick, MA). The directionality is Euclidean distance of cell movement divided by net distance along cell trajectory, which is calculated as 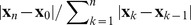. Here **x**_k_ is k^th^ cell position (k = 0, …, n), thus **x**_0_ and **x**_n_ represent initial and final cell positions. The time interval of the calculation (time interval of **x**_k-1_ and **x**_k_) is set as 1 min. The upward directionality is the directionality toward chemoattractant, which is defined as 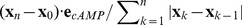, where **e**_cAMP_ means the unit vector pointing in the direction of positive chemoattractant gradient. The directional change is change of migration direction per unit time (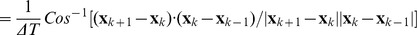), where ΔT is the time interval. Roundness is defined as 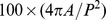, A and P represent area and perimeter of cell, respectively. The statistical significance was calculated by Student's t-test.

### qRT-PCR

A×4 cells were allowed to develop on 1% agar in KK2 buffer with or without 3 mM EGGG. Cells were harvested at each developmental time point, and total RNA was extracted using the RNeasy plus mini kit (QIAGEN). Prior to reverse transcription, the quality of RNA was checked using a chip-based analyzer (RNA 6000 Nano, RNA BioAnalyser 4000; Agilent Technologies). Two micrograms of total RNA was used as templates for cDNA synthesis using random hexamers and SuperScript III (First-Strand Synthesis; Invitrogen). qPCR amplification was performed using premixed solution of polymerase, UNG and dNTPs (TaqMan Universal PCR Master Mix; Applied Biosystems), primer pairs and TaqMan MGB probes listed in Table S1. Amplification reactions were monitored with the ABI7500 (Applied Biosystems), and results were analyzed using the comparative C_T_ method with *rnlA* gene amplification as an endogenous control.

### X-gal and Neutral Red Staining

A×4 cells were transformed with a plasmid containing a *pspA* promoter *lacZ* fusion (a kind gift from Dr. Jeff Williams and Dr. Tamao Saito) by electroporation following a standard protocol [28]. The obtained transformant *[pspA]:lacZ/A*×*4* cells were cultured in a modified HL-5 medium supplemented with 30 μg/ml of G418. 7×10^6^ cells were harvested and washed twice in KK2 buffer and developed on a nitrocellulose filter (2.2 cm in diameter; HAWP04700, Millipore) mounted on a 1% agar in KK2 with or without 3 mM EGCG at 22°C. The filter pad was collected and the sample was fixed with 0.5% glutaraldehyde and 0.1% Triton-X in Z-buffer (60 mM Na_2_HPO_4_, 40 mM NaH_2_PO_4_, 10 mM KCl, 1 mM MgSO_4_). The fixed cells were then bathed in staining solution (5 mM K_4_[Fe(CN)_6_], 5 mM K_3_[Fe(CN)_6_], 5 mM X-gal in Z-buffer) for 30 minutes at 37°C followed by destaining with Z-buffer as described [29]. For neutral red staining [30], growing A×4 cells were washed twice in KK2 buffer and resuspended at 2×10^7^ cells/ml in KK2 containing 0.005% neutral red. After incubation on ice for 10 minutes, cells were washed three times in KK2. 7×10^6^ cells were plated in each 3.8 cm^2^ well on a 12-well plate (3512, Corning) containing 1% agar in KK2 with or without 3 mM EGCG. Cells were incubated at 22°C and allowed to develop. Multicellular structures were observed under a dissecting microscope (SZX12, Olympus) and images were captured by a digital camera (CAMEDIA C-5060; Olympus).

## Results

**Figure 1:**
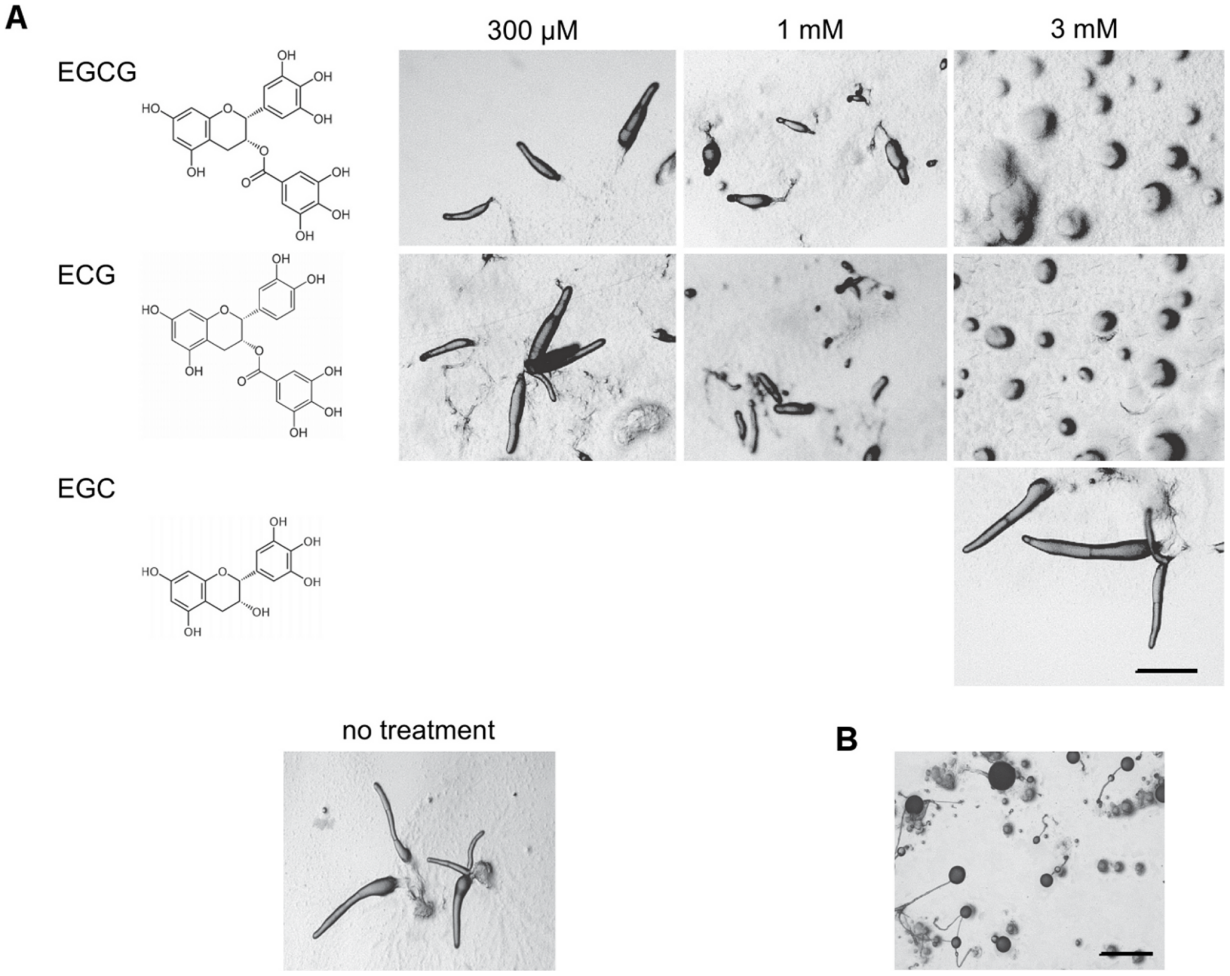
Effects of catechins on the Dictyostelium life cycle. *(*A) Amoebae were scraped from the feeding fronts of plaques formed on bacterial lawns and plated with increasing amounts of epigallocatechin (EGC), epicatechingallate (ECG) or epigallocatechin gallate (EGCG) and allowed to develop for 18 h before imaging with stereomicroscopy. (B) Mounds formed 18 h after plating with 3 mM EGCG were disaggregated by pipetting, plated on agar without catechin and incubated for 24 h. Bars, 1 mm.

To determine the effects of green tea catechins on the *Dictyostelium* life cycle, amoebae were plated on agar containing increasing amounts of epigallocatechin (EGC), epicatechin gallate (ECG) or epigallocatechin gallate (EGCG) (Figure 1). Under control conditions, cells aggregate into mounds that transition to finger and slug stages within 18 h of plating and go on to form typical fruiting bodies. In the presence of 3 mM ECG or EGCG aggregates form within 18 h, but they fail to progress to the finger stage of development (Figure 1A). Cells plated with 1 mM ECG or EGCG form small slugs and fruiting bodies while no discernable effects are seen in 300 μM of either compound. Aggregation and development are typical at 3 mM (Figure 1A) and 8 mM EGC (data not shown). EGCG is the best characterized and most potent green tea catechin, and we have focused our work on further characterizing the effects of this compound. To assess the possibility that morphogenesis is stalled due to cytotoxic effects of EGCG, aggregates that formed in wells containing 3 mM EGCG were disaggregated and plated on agar without EGCG. These cells begin to aggregate and develop within a few hours of replating, and fruiting bodies containing viable spores form within 24 h (Figure 1B). Furthermore, over eighty percent of amoebae isolated from aggregates after 48 h in the presence of EGCG stain positively with the vital dye fluoresceine diacetate, and new cultures can be initiated from aggregates that had continually been exposed to EGCG for 7 d (data not shown), suggesting that the effects of EGCG are not due to cytotoxicity.

**Figure 2:**
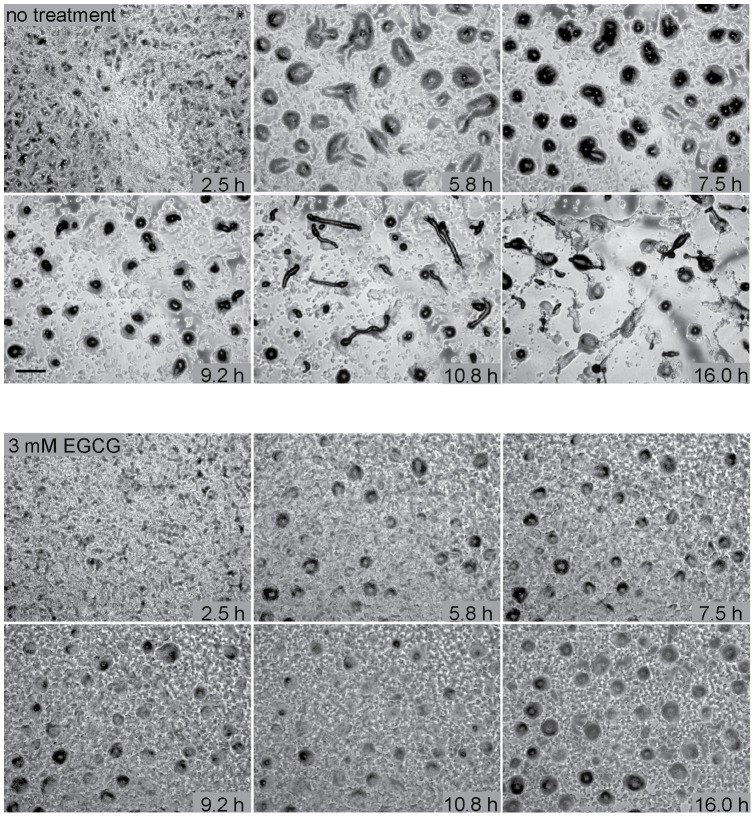
Time course of Dictyostelium development in the presence of epigallocatechin gallate. Amoebae grown in shaking axenic culture were plated on agar with or without 3 mM EGCG. Aggregation and development were followed for 13 h using time-lapse phase-contrast microscopy. Bar, 1 mm. Time-lapse movies are also available (Movie S1).

We also observed the kinetics and mechanism of aggregation using time-lapse microscopy. Under normal laboratory conditions, starving cells begin to aggregate within 5–6 h. Aggregating cells adhere front to back, forming streams that move towards the position where aggregates ultimately form (Figure 2, Movie S1). Aggregates form motile slugs at 10–12 h that migrate before culminating to form mature fruiting bodies at approximately 18–20 h post plating (data not shown). In cells incubated with 3 mM EGCG, timing of the onset of aggregation is typically similar to that seen with no treatment, although delays of 1–2 h are sometimes observed. Streams do not form in the presence of EGCG, though. Individual cells coalesce to form loose aggregates without aligning head to tail and large numbers of cells are left behind. Aggregates normally transition through two stages. In loose aggregates, cellular collections are generally flat with irregular edges. Loose aggregates transition into tight mounds, dome-like structures that are covered with cellulose-containing sheaths. In most experiments aggregates with the characteristics of loose aggregates formed (Figure 2, Movie S1). Aggregates never formed slugs, but mounds were occasionally capable of movement and motile aggregates moved as much as 1–2 mound diameters within 18–24 h.

**Figure 3:**
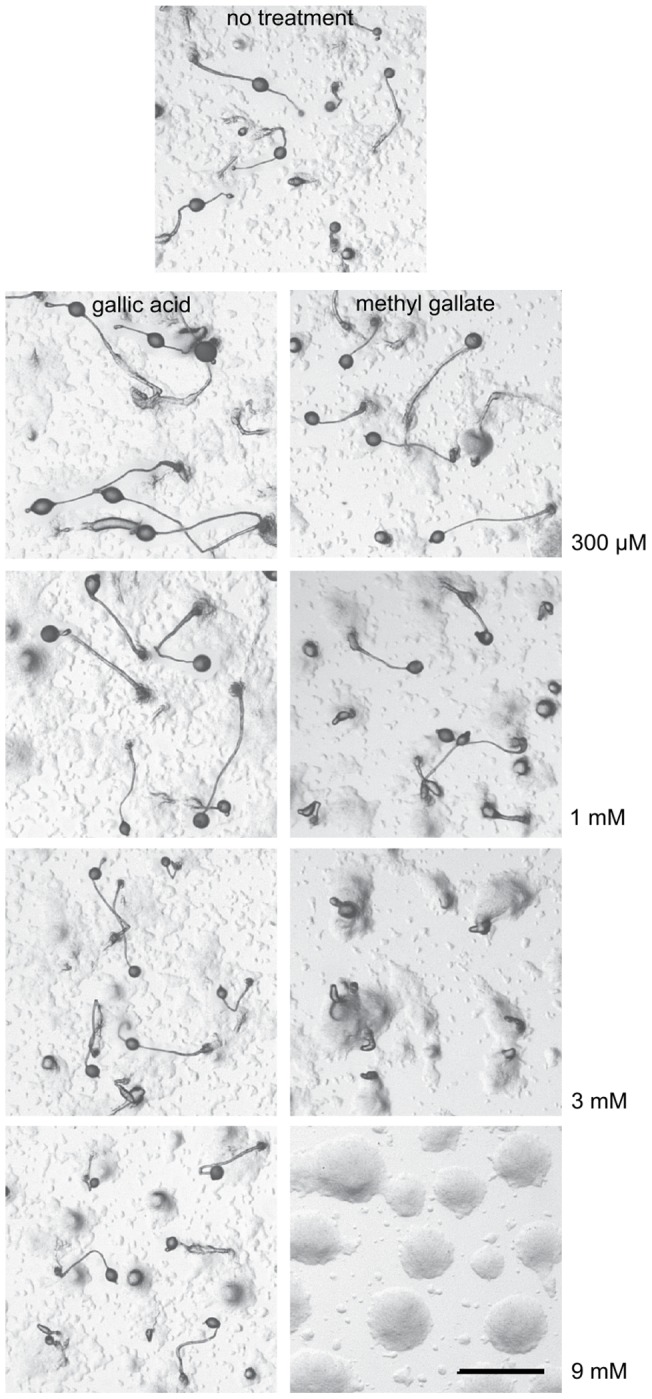
Effects of gallic acid and methyl gallate on Dictyostelium development. Cells were plated in the presence of increasing amounts of gallic acid or methyl 3,4,5-trihydroxybenzoic acid (methyl gallate) and allowed to develop for 18 h before imaging with stereomicroscopy. Bar, 1 mm.

Catechins share a fundamental arrangement of two benzene rings linked with a dihydropyran heterocycle. The catechin gallates ECG and EGCG both contain additional galloyl moieties substituted on the carbon 3 of the heterocycle. To characterize the structural requirements for inhibition, monolayers of amoebae were plated in increasing amounts of gallic acid or the gallate methyl ester, methyl 3,4,5-trihydroxybenzoate (Figure 3). While gallic acid has no effect at concentrations as high as 9 mM, 3 mM methyl gallate alters aggregation and blocks development. Cells aggregate without streaming and do not proceed through morphogenesis. Aggregates form probing tips that extend upwards and in some experiments away from mounds, but a typical slug is never formed and the aggregate does not move from where it is formed. Cells aggregate to form broad, shallow mounds in the presence of 9 mM methyl gallate. These data are consistent with the idea that the galloyl groups found in EGCG and ECG are required for the actions of these compounds.

**Figure 4:**
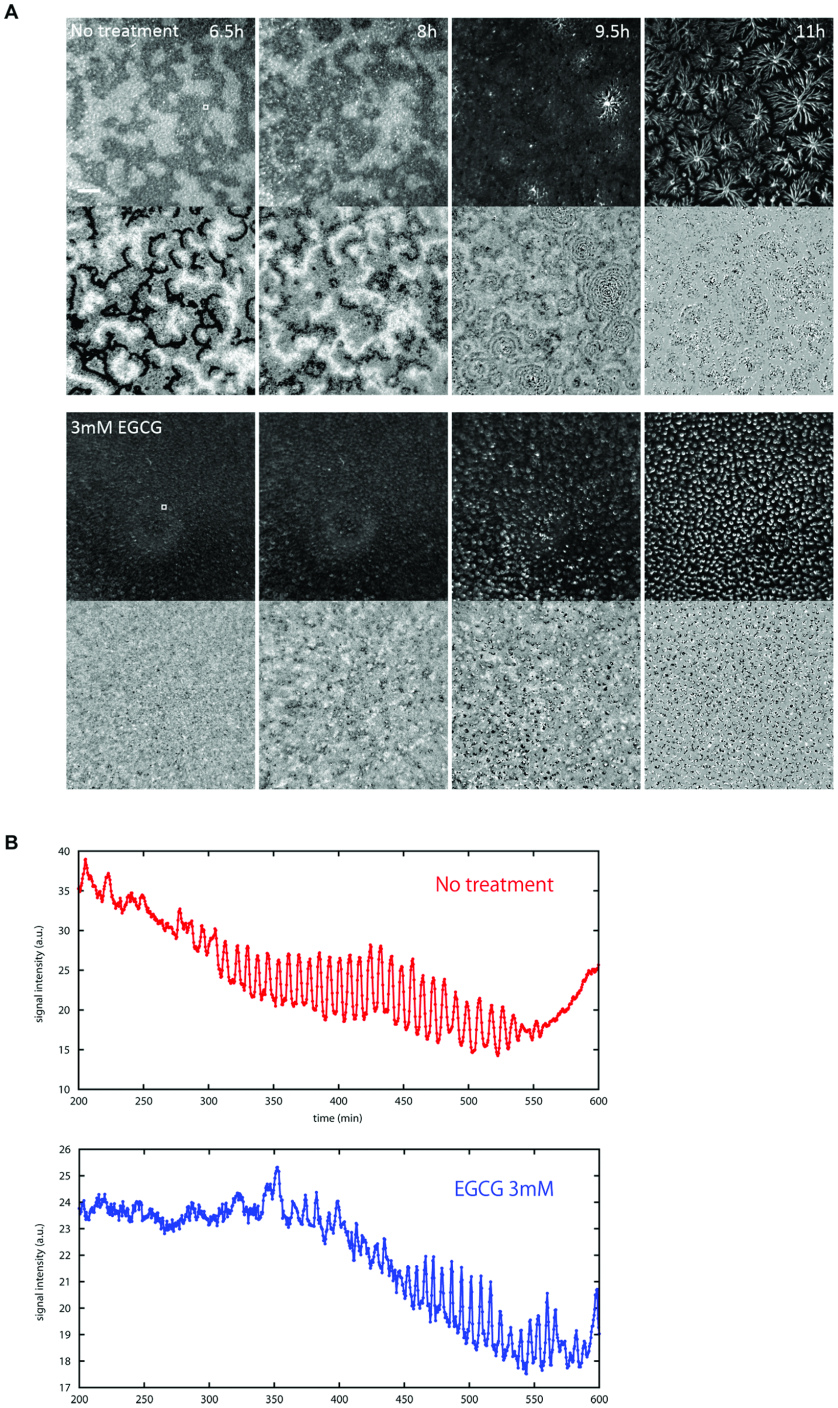
cAMP signaling in the presence of epigallocatechin gallate. Propagation of optical density waves observed under dark-field optics. (A) Snapshots at 6.5, 8, 9.5 and 11 h are shown for cells with/without EGCG. Propagating waves became clear by subtracting two successive (60 sec interval) images (lower panels). Scale bar is 2 mm. (B) Changes in the optical-density intensity were obtained from selected regions of interests (white box at 6.5 h images in (A)). Time-lapse images are also available (Movie S2).

To aggregate and develop, *Dictyostelium* amoebae must relay a chemotactic signal. In starved cells, secretion of the chemoattractant cAMP oscillates with a period of approximately six minutes; the secreted cAMP diffuses to activate neighboring cells to move towards the cAMP source and to secrete their own chemoattractant [31]–[32]. In a monolayer, propagation of these oscillations can be observed using darkfield optics, where cAMP responsive cells appear as light bands that move as concentric or spiral waves across the surface [21]. These bands reflect the shape changes that cells undergo when they respond to cAMP. Cells in light bands have received cAMP and are elongated and moving towards the cAMP source, while dark bands consist of less motile cells. To assess whether the aggregation defects observed in the presence of EGCG might be caused by aberrant propagation of cAMP waves, we observed cAMP waves in monolayers plated in the presence of EGCG (Figure 4). Cells treated with EGCG are able to propagate waves and these waves have a periodicity of 6–10 minutes, as is seen in control conditions. There are significant differences, however. First, wave propagation is delayed in the presence of EGCG. Waves are visible 4 hours after plating cells without EGCG, but waves are not seen until 5 hours after plating in the presence of the catechin. The waves that are generated are concentric and propagate short distances, and many signaling centers form. Finally, the amplitude of the waves that are generated is reduced suggesting that cAMP signal relay or the magnitude of cell shape change is muted by EGCG.

**Figure 5:**
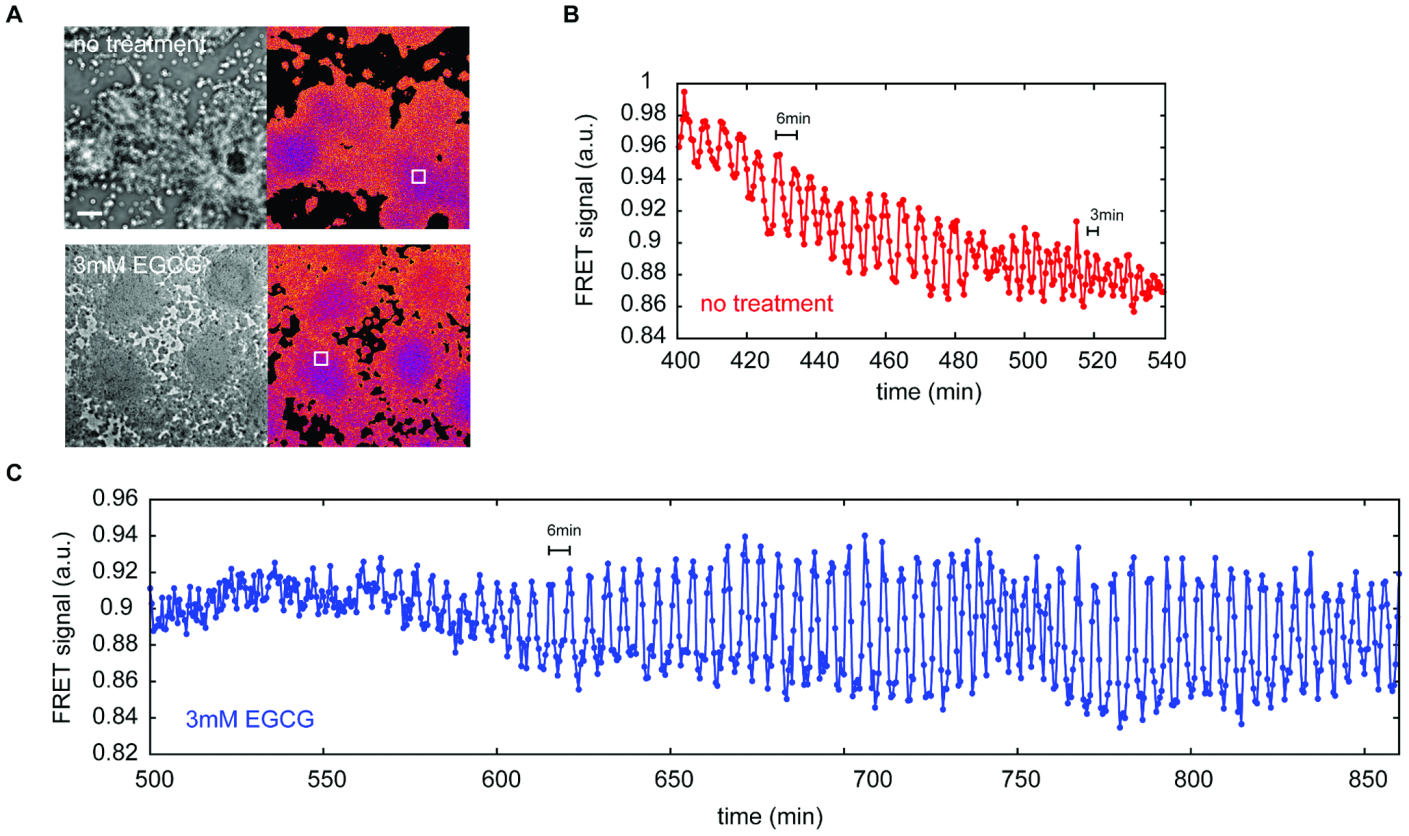
Effects of EGCG treatment on cAMP signaling in aggregates. The development of A×4 cells transformed by FRET sensor Epac1camps are observed. (A) Snapshots of developing cells with/without EGCG. Images of bright field (left) and FRET signal (right) were obtained at 8 h (no treatment) and 11 h (3 mM EGCG). Scale bar 100 μm. (B,C) Changes in FRET intensity with/without EGCG were obtained from selected regions of interests (white box in (A)). (B) Time course of FRET signal change for no treatment. The oscillation period shortens from 6 min to 3 min during the later stage of cell aggregation. (C) Time course of FRET signal change for 3 mM EGCG treatment. Oscillations with 5 to 6 min period are sustained for more than 5 h. For a brief moment at 730–760 min, oscillation period shortened before returning to 6 min. Fast oscillations seem to be unstable under the effects of EGCG. Time-lapse images are also available (Movie S3, S4).

In standard laboratory strains, optical density waves disappear during streaming [15] [33] presumably due to lack of morphological change by the aggregating cells during this stage of development. Although frame subtraction of bright field images and use of mutant strains have shown evidence of wave propagation during later stages of aggregation [34]–[35], data are subject to interpretation due to the fact that the observed periodicity is a combination of that inherent to cell movement and that from the cAMP oscillations. To directly assess the effects of EGCG on cAMP signaling in the aggregate, we used the FRET probe epac-1-camps (Figure 5), which is a readout for intracellular cAMP produced in response to extracellular cAMP waves [23]. Under typical conditions, changes in the level of cytosolic cAMP mimics what has been described for optical waves in early aggregates. Initial cAMP oscillations in non-treated cells have a period of 6 min and propagate as spiral waves across the entire aggregate (Figure 5B). The period shortens to approximately 3 min from the onset of cell streaming to early mound stage before the oscillations disappear during tip formation. In the presence of EGCG, cAMP oscillations are also observed but their onset is delayed for 2.5 h, consistent with our observations of optical density waves (Figure 5C). The oscillations persist for several hours at 6 min periodicity without making the transition to 3 min period oscillations. These waves usually take circular form, however they do not develop into multiple concentric wavefronts because signaling centers do not persist from fixed locations. A wavefront initiated from one signaling center is often superseded by a wavefront from newly occurring signaling centers; thus the continuous competition between the signaling centers prevent formation of large aggregation territories. Some spiral waves are also seen during this process. These observations suggest that cAMP relay that mediates directed cell streaming during the late aggregate to early mound stage is impaired by EGCG treatment.

**Figure 6:**
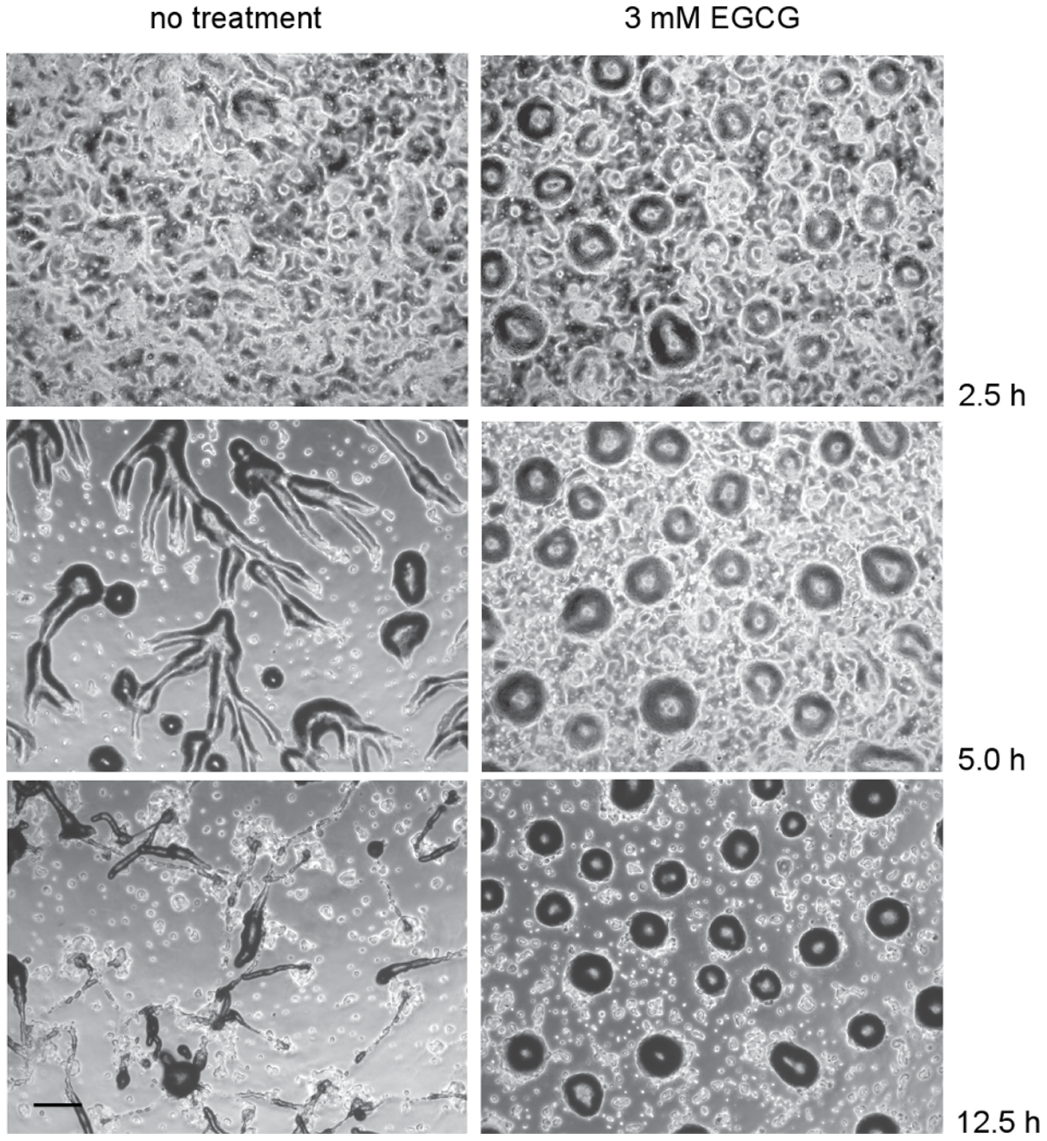
Aggregation of artificially-developed cells in the presence of EGCG. Axenically grown cells were resuspended in KK2 buffer, starved for 1 h and then pulsed for 5 h with 50 nM cAMP before plating on agar with and without 3 mM EGCG. Bar, 1 mm.

Aberrant wave propagation can result from defects in cAMP release, recognition or response. Pulsing cells with exogenous cAMP can sometimes rescue aggregation and developmental defects under conditions of defective cAMP relay by enhancing expression of genes normally induced by cAMP signaling [36]–[37]. Gene expression changes in cells that are pulsed with cAMP are similar to amoebae that have progressed approximately 12 h through development; the major genes required for aggregation and the initial stages of multicellular life are induced. To test whether exogenous cAMP rescues aggregation and morphogenesis, we plated cAMP-pulsed cells in the presence of EGCG (Figure 6). Aggregation in the presence of EGCG is accelerated with pulsing, with mounds forming within 2.5 h of plating. Pulsing does not rescue streaming or morphogenesis, however.

**Figure 7:**
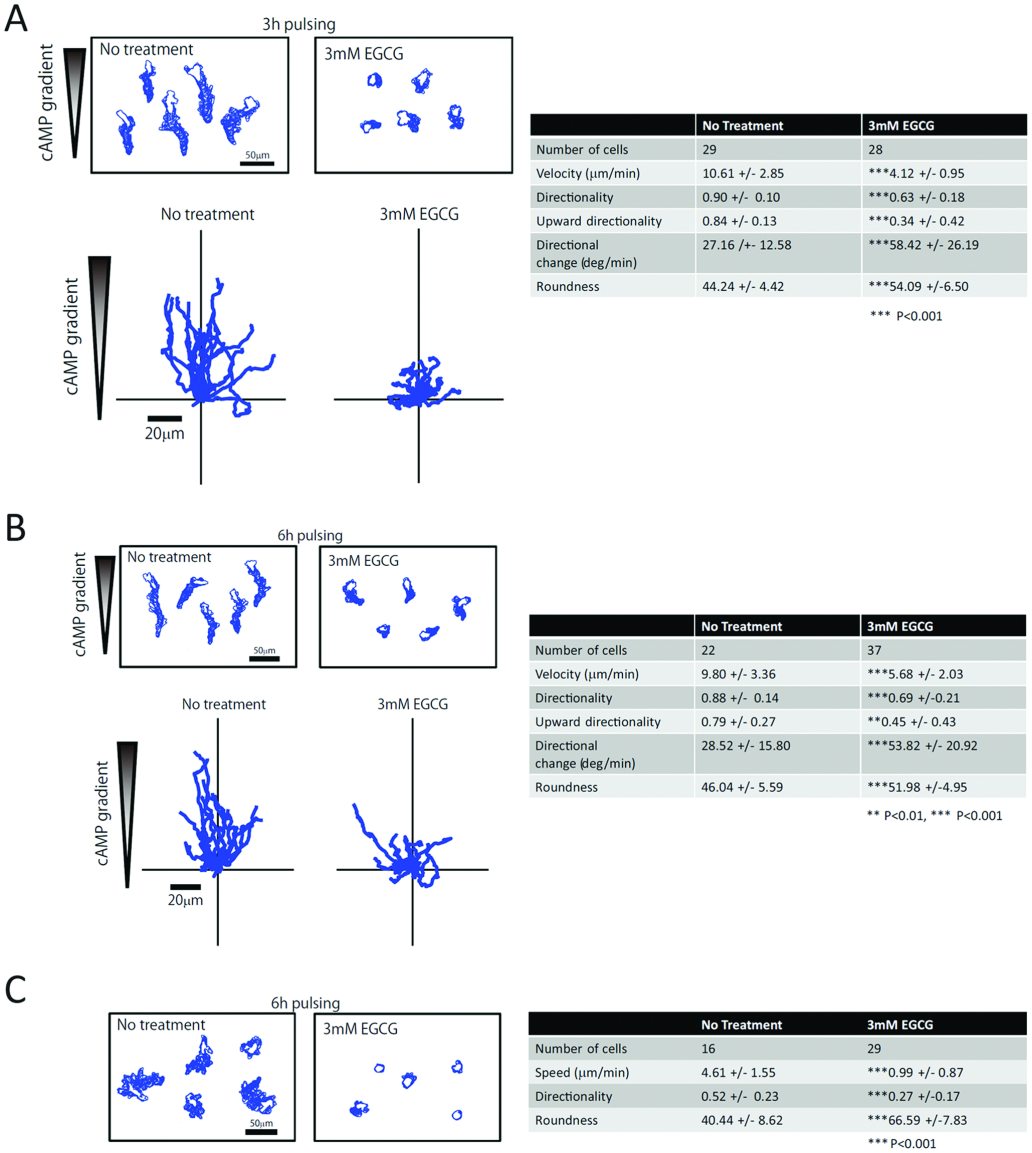
EGCG reduces cell motility and chemotaxis. Chemotaxis towards cAMP assessed in cells pulsed with cAMP for 3 h (A) or 6 h (B) and plated in Dunn chambers in the absence or presence of 3 mM EGCG. Outlines of individual motile cells are depicted in the upper left while cell trajectories are seen in the lower left. (C) Outlines of cAMP-pulsed cells plated without chemoattractant with or without EGCG. Quantitative analysis of cell motility is summarized in tables at the right of each panel.

The observations that cAMP waves are impaired during the late stage of aggregation where extracellular cAMP accumulates to micromolar range and that many cells are left behind and do not join the aggregate suggest that motility of cells under elevated extracellular cAMP concentration may also be affected by EGCG. To characterize potential motility defects, we monitored cAMP-dependent cell migration using a Dunn chamber assay in which cAMP-pulsed amoebae are exposed to a fixed gradient of cAMP. Cells that were pulsed for 3 h with exogenous cAMP exhibit many defects when plated in the presence of 3 mM EGCG (Figure 7A). Although many EGCG-treated cells move towards cAMP, upward directionality, a measure of motility in the direction of the gradient is significantly reduced, suggesting that gradient sensing is affected. Amoebae also move more slowly, exhibiting a 60% reduction in velocity when compared to untreated cells. The route they take is less direct and cells turn often, with increases in directional change and a decrease in directional persistence. Finally, cell roundness is increased, signifying that pulsed cells do not elongate typically when treated with EGCG. Increasing the duration of pulsing to 6 h appears to moderate these effects, but motility metrics do not reach levels seen without catechin (Figure 7B). We also assayed random motility of cells that were pulsed 6 h and plated in buffer lacking chemoattractant (Figure 7C). In the absence of EGCG, cells extend numerous pseudopodia and translocate several cell diameters over the 15 min of observation. Cells plated in EGCG extend fewer pseudopodia and do not elongate or translocate across the substratum.

**Figure 8:**
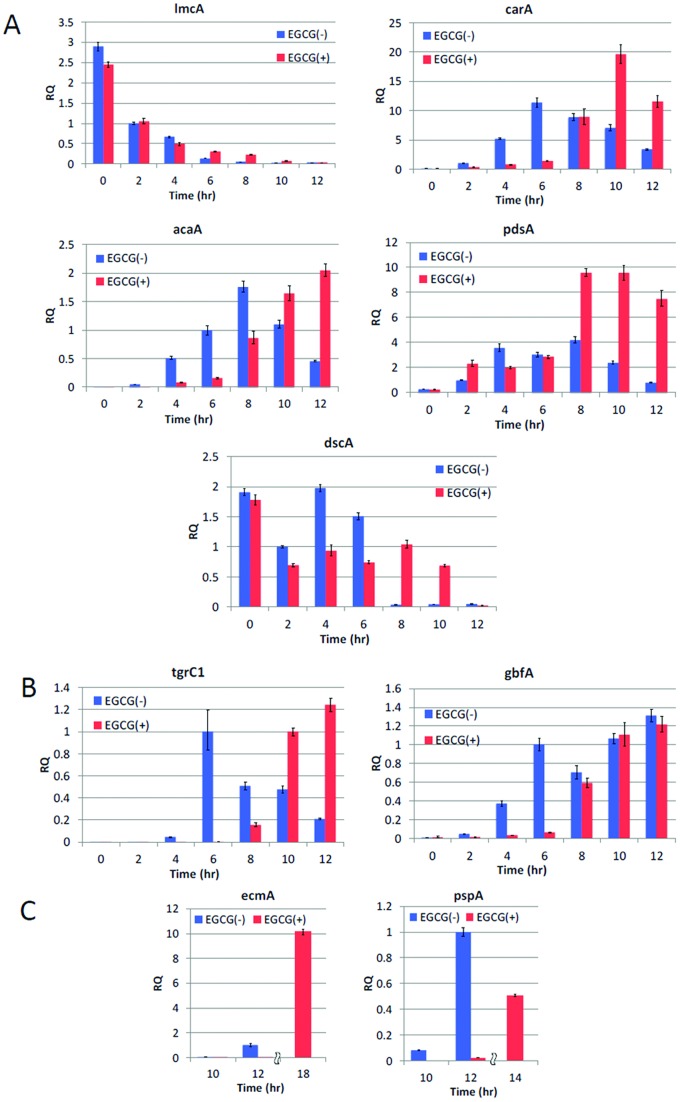
Effects of EGCG on the expression of developmentally-regulated genes. Relative expression level of genes that work mostly during: (A) transition from growth to development, cAMP signaling and cell aggregation; (B) late aggregate to first finger; and (C) prestalk and prespore cell differentiation. Data are obtained for 0–12 h after starvation ((A) and (B)) and 10 and 12 h (C). In addition, expression level of *ecmA* and *pspA* at 18 h and 14 h respectively are shown for EGCG treated cells in (C). Expression levels were quantified using the comparative C_T_ method using amplification of *rnlA* gene as an endogenous control.

The aggregation and developmental stages affected by EGCG rely on gene expression changes that are first induced by nutrient depletion and further regulated by cAMP signaling. To determine if EGCG alters developmentally regulated gene expression in *Dictyostelium*, we monitored mRNA levels of several genes involved in the aggregation phase of the *Dictyostelium* life cycle using quantitative real-time PCR. We found that in the presence of EGCG expression of the vegetative stage gene *lmcA* is downregulated normally while the aggregation stage genes *carA*, *acaA*, and *pdsA* essential for cAMP signaling and the *dscA* gene that is a marker for early development are delayed for 4–6 h (Figure 8A). The delays in expression were also observed for *tgrC* and *gbfA,* genes expressed from late aggregate to early mound stage (Figure 8B). These observations are consistent with delayed onset of cAMP waves observed in EGCG treated cells. For cell differentiation markers, we detected almost no expression of prespore specific *pspA* and prestalk specific *ecmA* genes in EGCG treated cells at 10 to 12 h into starvation, the time at which differentiated cells first appear and segregate to form tipped mounds under control conditions (Figure 8C). These genes were expressed at 14–16 h after starvation.

**Figure 9:**
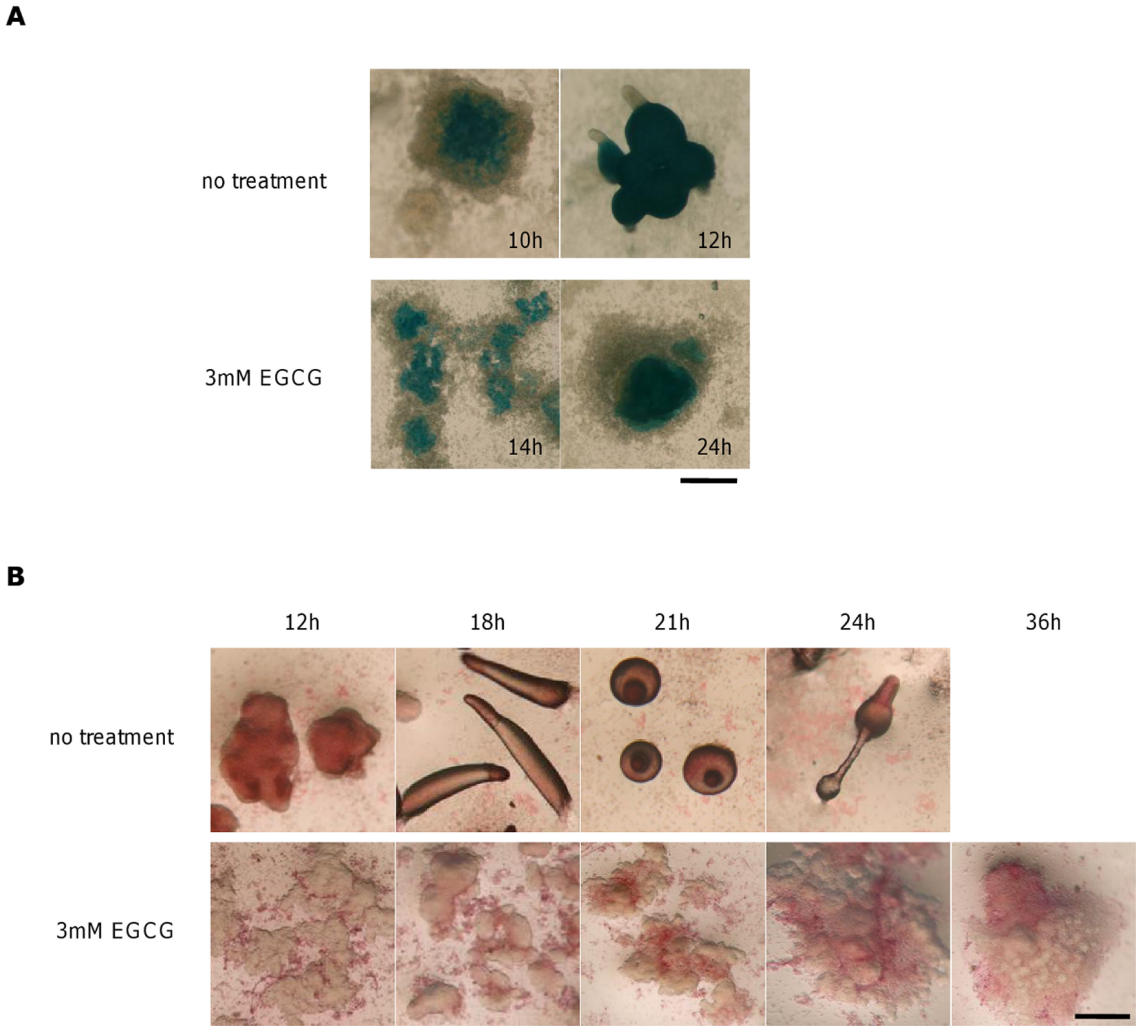
Effects of EGCG on cell sorting of differentiating cells. (A) X-gal staining of A×4 cells expressing *lacZ* under the prespore-specific *pspA* promoter. Without EGCG (upper panels) and with EGCG (lower panels). Scale bar: 0.5 mm. (B) Neutral red staining of A×4 cells. Without EGCG (upper panels) and with EGCG (lower panels). Scale bar: 0.4 mm.

Furthermore, we stained for cells expressing the *pspA* gene using cells expressing *lacZ* under the *pspA* promoter. For non-treated cells, the early appearance of *pspA* expressing cells is somewhat scattered but more concentrated in the center of the mound (Figure 9A; top 12 h) as previously reported [38]. For EGCG treated cells, this pattern was delayed by 2–4 hours (Figure 9A; bottom 14 h) consistent with the qPCR analysis. In the presence of EGCG, the prespore cells remained in this spatial configuration for more than 12 h (Figure 9A; bottom 24 h), while in untreated cells many of the mounds underwent morphogenetic movement and formed the first fingers where the stained prespore cells appear behind the unstained prestalk cells at the top (Figure 9A; top 12 h). To observe prestalk cells, we employed the vital stain neutral red that marks the acidic autophagic vacuoles of the prestalk cells [39]. In non-treated cells, neutral red stained cells first appeared throughout the mound with somewhat higher intensity either in the periphery or the tip region of the mound (Figure 9B; top 12 h). The stained cells then became more clearly confined to anterior 1/4 of the migrating slugs and a few scattered anterior-like cells in the posterior region and the rear guard cells at the posterior end (Figure 9B; top 18 h). At the Mexican hat stage (Figure 9B; top 21 h), stronger neutral red staining was observed at the tip but the difference became difficult to see in the culminants (Figure 9B; top 24 h) due to vacuolation of stalk cells. In the presence of EGCG, neutral red positive cells appeared scattered in the aggregates (Figure 9B; bottom 12 h). These cells slowly coalesced (Figure 9B; bottom 18 and 21 h) and localized to the peripheral region of the mounds (Figure 9B bottom 24 h). The neutral red positive cells eventually segregated and formed a larger cell mass (Figure 9B; bottom 36 h). These patterns contrast well with the opposite staining patterns of prespore cells and together demonstrate deficiency in prestalk/prespore cell sorting in EGCG. These observations indicate that although cell differentiation takes place in the presence of EGCG, morphogenetic cell movement that transforms the mound to the first finger is impaired.

## Discussion

The green tea catechin EGCG has multiple effects on the early stages of the *Dictyostelium* life cycle. Effects are seen at high concentrations relative to the micromolar levels typically used in mammalian cells, possibly because EGCG is less effective in *Dictyostelium* or because EGCG is expelled from cells through a robust system of ABC transporters expressed in this organism [40]. Treatment is reversible and levels of EGCG used are not remarkably toxic. The effects appear to be mediated by a galloyl group that is found in EGCG and ECG and has been shown to inhibit a number of enzymes including collagenase [41], fatty acid synthase [42] and pancreatic lipase [43] in addition to being important for anti-viral [44]–[45] and anti-inflammatory activities [46].

The *Dictyostelium* life cycle relies on changes in gene expression [36], [37] that could be affected by EGCG. Upon nutrient depletion, cells induce expression of genes that are necessary for initiation of cAMP relay and chemotaxis (e.g., *carA*, *acaA*, *pdsA*). cAMP signaling induces further changes in expression of these genes involved in the aggregation stage and others in the late aggregate that are involved in multicellularity, differentiation and morphogenesis (e.g., *tgrC1*, *gbfA*, *ecmA*, *pspA*) [47], [48], [49]. Expression of these developmentally-regulated genes is delayed 4–6 h in the presence of EGCG, but expression eventually approaches levels seen under control conditions and differentiated cells are seen in mounds that formed in EGCG. Finally, pulsing with exogenous cAMP, which induces expression of these genes [36], rescues neither streaming nor morphogenesis. Together, these data suggest that the effects of EGCG are not primarily due to altered gene expression, although we cannot exclude the possibility that misexpression of genes not assessed here contributes to the observed phenotypes.

Chemotaxis towards cAMP is required for aggregation leading to multicellularity in *Dictyostelium*. During aggregation cAMP pulses result in cell shape changes that are associated with motility and can be observed using dark-field optics. In EGCG, emergence of dark-field waves is delayed by approximately 1 h, a finding that may reflect the delay in expression of genes involved in aggregation. Once initiated, waves propagate short distances, resulting in small aggregation territories, and the resulting waves have reduced amplitude. Cells do not stream and many amoebae fail to aggregate. These phenotypes suggest defects in chemotaxis towards cAMP. Indeed, motility is significantly impaired in EGCG. When placed in a spatial gradient of cAMP, EGCG-treated cells fail to elongate normally and move slowly. Their sense of direction is also less acute. These effects are reminiscent of those seen with a loss of PI3K activity. Cells in which PI3K activity is reduced by gene knockout or inhibition move up shallow fixed cAMP gradients [50]–[51], but do so with defects in directionality and cell shape similar to those seen here, albeit in steeper gradients. Pulsing these cells with exogenous cAMP moderates motility defects [50], [52], a result that is again consistent with the effects we see here with EGCG. Furthermore, loss of PI3K blocks streaming during aggregation [53]. These results, taken together with the observation that EGCG blocks PI3K activity in mammalian tumor cell lines [54], suggest that EGCG might be acting through this signaling protein. It will be interesting to determine if its effects result from inhibition of PI3K or other targets.

Morphogenesis at the mound stage also requires chemotaxis towards cAMP [55]–[58]. Prespore and prestalk cells self-associate, possibly due to a combination of differential adhesion and chemotaxis, although the precise mechanisms remain unclear. Prestalk cells then move in a cAMP-dependent fashion towards the top of mounds, where they form tips that direct further morphogenesis. In the presence of EGCG, prespore cells eventually segregate to the center of mounds, as is seen under control conditions. Prestalk cells also self-associate, but slowly, and cell masses appear at the periphery of the mound. It appears that homotypic cell adhesion occurs in mounds but that motility known to be necessary for apical sorting of prestalk cells is impaired, a result that is consistent with the chemotaxis defects seen in our Dunn chamber experiments. An alternate possibility is that EGCG blocks formation of the cAMP gradient that is needed for prestalk cell motility leading to tip formation. A sorting pattern similar to what is seen here with EGCG has been described when a section of a differentiating slug is placed on agar containing cAMP, a process that is thought to prevent apical-basal cAMP gradient formation [55].

Work described here demonstrates that EGCG has pleiotropic effects on the *Dictyostelium* life cycle, suggesting that this organism is a useful model in which to characterize biological effects of catechins. Furthermore, catechins and their derivatives may be useful for investigating chemotaxis necessary for aggregation and morphogenesis in this organism. Finally, the methods used here to characterize development are powerful tools to investigate the *Dictyostelium* life cycle. Most are relatively simple, quick, automated and easily adaptable for high-throughput screening. These features suggest *Dictyostelium* to be a useful model in which to identify and characterize the activities of other bioactive compounds.

## Supporting Information

Movie S1 *Effects of EGCG on Dictyostelium aggregation and development.* Movie highlighting EGCG effects described in Figure 1. Phase contrast images were acquired every minute and compiled into .avi files using MetaMorph. The resulting movies were combined in a single panel using Adobe Premiere. Duration of the movie is 16 h.
(AVI)

Movie S2 *Effects of EGCG treatment on cAMP signaling.* The time-lapse acquired under dark-filed optics (snapshots are shown in Figure 4). Images were acquired every 30 seconds. Optical density waves were highlighted by image subtraction of successive images (1 min interval) (lower panels). Duration of the movie is 13 h.
(MOV)

Movie S3 *cAMP signaling during aggregation without EGCG treatment.* Spatio-temporal cAMP propagation were obtained by FRET signals of cAMP indicator Epac1-camps. FRET signal intensities are indicated as heat map (a snapshot is shown in Figure 7A). High concentration of cAMP is indicated by yellow and low concentration of cAMP is indicated by purple. Images were acquired every 30 seconds and the duration of the movie is 140 min. The field of view is 0.8 mm ×0.8 mm.
(MOV)

Movie S4 *cAMP signaling during aggregation under 3mM EGCG* (a snapshot is shown in Figure 7B). Images were acquired every 30 seconds and the duration of the movie is 360 min. The field of view is 0.8 mm ×0.8 mm.
(MOV)

Table S1 *Oligonucleotides used for qRT-PCR analysis.*
(DOCX)
