# Supplementary material for: The Green Tea Catechin Epigallocatechin Gallate (EGCG) Blocks Cell Motility, Chemotaxis and Development in Dictyostelium discoideum
Source: PLoS One. 2013 Mar 14;8(3):e59275. doi: 10.1371/journal.pone.0059275 (PMC3597604; doi:10.1371/journal.pone.0059275)
Supplement: Table S1 — Oligonucleotides used for qRT-PCR analysis. (DOCX) [file pone.0059275.s005.docx]

**Table S1:** Primers used for qRT-PCR

|  |  |  | ATG = +1 | |
| --- | --- | --- | --- | --- |
|  |  | 5' →3' | start | end |
| *lmcA* | Forward | CCTTGGCTGCTACCAATAACG | 116 | 136 |
|  | Reverse | CTTTTTCTGGCATTTGTTATGTAATTAGG | 160 | 188 |
|  | TaqMan probe | TGCCAATTATGATTACGCC | 138 | 156 |
| *carA* | Forward | AAATATGTTTCCACCAGCACTCAA | 693 | 716 |
|  | Reverse | GATAAATGTGACAGATGCCCAAAA | 751 | 774 |
|  | TaqMan probe | ATTCTCCACACCTATTTG | 718 | 735 |
| *pdsA* | Forward | AGCAAGTGGCATTGAATATCCA | 789 | 807 |
|  | Reverse | ACCAAAGACATAGTGGTGGCATT | 829 | 851 |
|  | TaqMan probe | TCACAGAGTTGGTCCC | 809 | 824 |
| *acaA* | Forward | TTGGTATTAGTCATGGTCCTTTGG | 3833 | 3856 |
|  | Reverse | GAGGCGGTATTGGCAGTATCA | 3903 | 3923 |
|  | TaqMan probe | CTGGTTGTATCGGTATCAG | 3860 | 3878 |
| *dscA* | Forward | GGTCGTGGTGATGCTGATCA | 232 | 251 |
|  | Reverse | CGATATTCAAACCAGGAAACATTATC | 289 | 311 |
|  | TaqMan probe | TACATCATACAAAATCCG | 258 | 275 |
| *tgrC* | Forward | CCTCCAACACCAATAGATGCAA | 64 | 85 |
|  | Reverse | GTTCTGGGTTCTTTTTCGTTTTTATACA | 152 | 179 |
|  | TaqMan probe | TAATAGTAATCTCCCATATTCTACC | 117 | 141 |
| *gbfA* | Forward | TCTTTCTCAAATACTGGTGCACTTTT | 1924 | 1949 |
|  | Reverse | CGACACAACTAACCAATACTTGAACA | 2049 | 2074 |
|  | TaqMan probe | CAAGTAATGGAATTAATCTAGC | 1952 | 1973 |
| *ecmA* | Forward | GTTAATGCGGAAACTGAAACCA | 58 | 79 |
|  | Reverse | CAAAAAGTAAACCTGCAGAACACA | 146 | 169 |
|  | TaqMan probe | ACAAACCAATACAGCATGTG | 81 | 100 |
| *pspA* | Forward | GCGCTGATCAAACTTCTTCACAT | 263 | 285 |
|  | Reverse | GGGTGTGGCAGTGATTTTACAA | 312 | 333 |
|  | TaqMan probe | CACTCGGTTCTGATTGG | 287 | 303 |
| *rnlA* | Forward | CGGATAAAAGGTACGCTAGGGATA | 2327 | 2350 |
|  | Reverse | GTGCCGAACCACATAACAGATATG | 2375 | 2398 |
|  | TaqMan probe | CAGGCTAGTCACATATT | 2352 | 2368 |
